# On the distance of genetic relationships and the accuracy of genomic prediction in pig breeding

**DOI:** 10.1186/1297-9686-46-49

**Published:** 2014-08-01

**Authors:** Theo HE Meuwissen, Jorgen Odegard, Ina Andersen-Ranberg, Eli Grindflek

**Affiliations:** 1 Institute of Animal and Aquacultural Sciences, Norwegian University of Life Sciences, Ås, Norway; 2 Aqua Aqua Gen AS, P.O. Box 1240, Sluppen, Trondheim NO-7462, Norway; 3 NORSVIN (The Norwegian Pig Breeders Association), PO Box 504, Hamar 2304, Norway

## Abstract

**Background:**

With the advent of genomic selection, alternative relationship matrices are used in animal breeding, which vary in their coverage of distant relationships due to old common ancestors. Relationships based on pedigree (**A**) and linkage analysis (**G**_**LA**_) cover only recent relationships because of the limited depth of the known pedigree. Relationships based on identity-by-state (**G**) include relationships up to the age of the SNP (single nucleotide polymorphism) mutations. We hypothesised that the latter relationships were too old, since QTL (quantitative trait locus) mutations for traits under selection were probably more recent than the SNPs on a chip, which are typically selected for high minor allele frequency. In addition, **A** and **G**_**LA**_ relationships are too recent to cover genetic differences accurately. Thus, we devised a relationship matrix that considered intermediate-aged relationships and compared all these relationship matrices for their accuracy of genomic prediction in a pig breeding situation.

**Methods:**

Haplotypes were constructed and used to build a haplotype-based relationship matrix (**G**_**H**_), which considers more intermediate-aged relationships, since haplotypes recombine more quickly than SNPs mutate. Dense genotypes (38 453 SNPs) on 3250 elite breeding pigs were combined with phenotypes for growth rate (2668 records), lean meat percentage (2618), weight at three weeks of age (7387) and number of teats (5851) to estimate breeding values for all animals in the pedigree (8187 animals) using the aforementioned relationship matrices. Phenotypes on the youngest 424 to 486 animals were masked and predicted in order to assess the accuracy of the alternative genomic predictions.

**Results:**

Correlations between the relationships and regressions of older on younger relationships revealed that the age of the relationships increased in the order **A**, **G**_**LA**_, **G**_**H**_ and **G**. Use of genomic relationship matrices yielded significantly higher prediction accuracies than **A**. **G**_**H**_ and **G**, differed not significantly, but were significantly more accurate than **G**_**LA**_.

**Conclusions:**

Our hypothesis that intermediate-aged relationships yield more accurate genomic predictions than **G** was confirmed for two of four traits, but these results were not statistically significant. Use of estimated genotype probabilities for ungenotyped animals proved to be an efficient method to include the phenotypes of ungenotyped animals.

## Background

Wright’s [1] numerator relationship matrix, **A**, is based on pedigree relationships and relies on the assumption of a base population, in which animals are unrelated, i.e., without known parents and non-inbred. Relationship and inbreeding coefficients are expressed in terms of Identity-by-Descent (IBD) probabilities, where the IBD occurs after the base population was established. If the base population is moved further back in time, IBD probabilities increase and eventually approach 1. Inbreeding coefficients (F) and relationships should thus be evaluated relative to each other and not in terms of their absolute values. For instance, the rate of inbreeding, ΔF = (F_t_ - F_t - 1_)/(1 - F_t - 1_), expresses the difference in inbreeding between generations t and t-1 relative to the maximum level of inbreeding, and is robust to the choice of the base population. For practical reasons, base populations are usually quite recent, because old pedigrees may not be available, or are rather incomplete, or because numerator relationships (**A**) reduce quickly over generations and Best Linear Unbiased Prediction of Breeding Values based on **A** (ABLUP-EBV) are not much affected by information from old ancestors.

GBLUP-EBV are BLUP- estimated breeding values based on genomic relationship matrices, **G**, and are commonly used in genomic selection (GS) [2,3]. Genomic relationship matrices are based on alleles at molecular genetic markers being Identical-By-State (IBS). When tracing the inheritance of the two marker alleles back in time, their paths of inheritance eventually coalesce into a single common ancestor, and IBS thus implies that there was no mutation in any of these inheritance paths. Because of this (ancient) common ancestor, two alleles that are IBS are also IBD. Thus, marker-based IBS relationship matrices are also expressed relative to a base population, which is on average 1/(2ν) generations ago, where ν is the SNP mutation rate, as shown by [4] but considering recombination events instead of mutations. However, if the effective population size, N_e_, is small, the two paths coalesce rapidly, which implies that only recent mutations result in DNA polymorphisms (old mutations have either been fixed or lost in a small population). Thus, for small N_e_, a slightly higher mutation rate may be assumed in the 1/(2ν) term to mimic the young age of most mutations. Especially when the markers on the SNP panel were selected based on having high minor allele frequencies (MAF), the SNP markers reflect rather old mutations. This is because all mutations start at a low frequency, and most mutations are lost before reaching substantial allele frequencies. It follows that low MAF alleles are mainly due to young mutations and high MAF alleles represent quite old mutations. Thus, if markers are selected based on high MAF, marker mutations may well predate QTL mutations that affect traits of interest, because traits of interest have been under selection, and old mutations that affect them were either lost or fixed. In the case of disease resistance traits, natural selection may have weeded out deleterious alleles and existing genetic variation may be due to relatively recent mutations. Even for neutral loci, e.g. for a neutral trait, there will be relatively more low MAF genes than on the SNP-chip.

IBS between alleles at a locus for two gametes is strictly defined as the molecular coancestry, i.e. $f_{M_{\mathrm{ij}}}=x_{i}x_{j}+\left(1-x_{i}\right)\left(1-x_{\mathrm{ij}}\right)$, where x_i_ (x_j_) is the allele state code (x_i_ = 0 or 1) for gamete i (j) [5]. VanRaden’s [6] estimate of the genomic relationship is $g_{VR_{\mathrm{ij}}}=\left(x_{i}-0.5\right)\left(x_{j}-0.5\right)/0.25$, assuming allele frequencies of 0.5 in order to maximise expected relationships. Since $g_{VR_{\mathrm{ij}}}=2f_{M_{\mathrm{ij}}}-1$, these estimates are proportional to each other, and we will thus consider the resulting genomic relationship matrix, **G**, as indicating IBS relationships. IBS-based relationship matrices, as commonly used in GS, reflect rather old relationships, whereas pedigree-based relationships, **A**, are rather young and decay quickly. The latter may be improved by the use of relationship matrices based on genome-wide linkage analysis, G_LA_, which combine pedigree and marker information, which in dairy cattle have yielded similar accuracies as GBLUP [7]. However, linkage analysis relationships may be as young as pedigree relationships, or even younger when the base population is put forward in time due to lack of genotype data on old ancestors. Although **G** and **G**_**LA**_ relationship matrices yielded very similar accuracies [7], they contain quite different relationships, which suggests that intermediate-aged relationships may improve the accuracy of GS. Habier et al. [8] distinguished three sources of information for GS: (i) family relationships, as contained in the pedigree; (ii) linkage analysis information, as contained in **G**_**LA**_, which they called co-segregation of alleles; and (iii) linkage disequilibrium (LD) information, as contained in **G**, and which is already present in the base population. This distinction of information sources coincides well with our distinction of ages of the relationships, indicating that the relationships at different ages tend to reflect fundamentally different sources of information.

In view of this background, it seems that the **G** matrix traces relationships that are too old and **G**_**LA**_ traces only very recent relationships. Thus, we hypothesised that relationships of more intermediate age are more appropriate for GS, and developed a haplotype-based relationship matrix, **G**_**H**_, since recombination of haplotypes occurs more frequently than mutations at single SNPs. Our aim was to compare relationship matrices that express relationships over different genetic distances (ages), and the resulting accuracies of GS in a pig breeding situation.

## Methods

### Genotyping data

Genotyping and phenotyping data were kindly provided by Norsvin AS. Genotypes from 3250 Norwegian Landrace pigs were available, of which 2553 boars came from the boar-test station and 697 dams from the nucleus herds, all born between 2010 and 2013. All animals were genotyped at CIGENE (http://www.cigene.no), using the porcine 60 K SNP array from Illumina (Illumina, San Diego, CA, USA). Clustering and genotype calling were performed using the genotyping module in the Genome Studio software (Illumina, San Diego, CA, USA). In total, 60 451 SNPs were used for genotyping, and 38 453 informative markers passed quality control, which was based on having a MAF > 0.01, call frequency > 0.10, and parent–child Mendelian errors < 0.025. Samples were included in the analysis if their call rate was > 75%, although the average call rate was equal to 99.5% with a standard deviation of 1.6%. Parentage tests are routinely performed for all boars at the boar test station so no pedigree errors were observed. Occasional missing genotypes were imputed and the genotype data were phased using Beagle v3.3.1 [9]. After quality control, a total of 3250 genotyped animals were available for analysis. The pedigree of the genotyped animals was traced back for five generations to form a pedigree file containing 8187 animals.

### Phenotypic records

Four traits were chosen for analysis, mainly based on their high frequency of recording, such that the number of animals with missing phenotypes was relatively small. The traits were a mixture of production, product quality and reproduction traits: growth rate (GR), measured in number of days required to grow from 25 to 100 kg live weight; meat percentage (M%), which is measured on live evaluation of boars by computer tomography, using Norsvin’s image analysis software (Jørgen Kongsro, Norsvin, personal communication); weight at 3 weeks of age (W3W), which is mainly viewed as a fitness trait of the piglet; number of teats (NT), which is counted on both males and females. All available phenotypes on all 8187 animals in the pedigree were collected, including on non-genotyped animals. The number of phenotypes and trait heritabilities (as used in Norsvin’s routine breeding value evaluation) are in Table 1.

**Table 1:** Number of records and genetic parameters of the analysed traits: growth (GR), meat percentage (M%), weight at 3 weeks (W3W) and number of teats (NT)

	**GR**	**M%**	**W3W**	**NT**
	**Number of phenotypes**			
Total	2668	2618	7387	6851
Genotyped	2504	2472	3244	3225
Non-genotyped.	154	146	4143	3626
Masked	458	424	486	486
	**Variance components**			
Genetic	15.3	3.34	0.127	0.342
Residual	22.5	3.56	1.157	0.539
Litter	5	0.3	0.661	0.04
Pen	3.2	2.7	X	X
Heritability	0.40	0.48	0.10	0.39

### Estimation of breeding values

The data were analysed with single-trait animal models using statistical models that were the same as those used by Norsvin for their routine EBV estimation:

$$\begin{array}{l} y_{\mathrm{GR}}\;=\;F_{\mathrm{GR}}f_{\mathrm{GR}}\;+\;S_{\mathrm{GR}}s_{\mathrm{GR}}\;+\;L_{\mathrm{GR}}l_{\mathrm{GR}}\;+\;P_{\mathrm{GR}}p_{\mathrm{GR}}\;+\;Z_{\mathrm{GR}}a_{\mathrm{GR}}\;+\;e_{\mathrm{GR}}\\ y_{M\%}\;=\;F_{M\%}f_{M\%}\;+\;V_{M\%}v_{M\%}\;+\;wb_{M\%\;\mathrm{on}\;w}\;+\;L_{M\%}l_{M\%}\;+\;P_{M\%}p_{M\%}\;\\ \;+Z_{M\%}a_{M\%}\;+\;e_{M\%}\\ y_{W3W}\;=\;F_{W3W}f_{W3W}+S_{W3W}s_{W3W}+N_{W3W}n_{W3W}+D_{W3W}d_{W3W}\;\\ \;+L_{W3W}l_{W3W}\;+\;Z_{W3W}a_{W3W}\;+\;e_{W3W}\\ y_{\mathrm{NT}}\;=\;F_{\mathrm{NT}}f_{\mathrm{NT}}\;+\;S_{\mathrm{NT}}s_{\mathrm{NT}}\;+\;L_{\mathrm{NT}}l_{\mathrm{NT}}\;+\;Z_{\mathrm{NT}}a_{\mathrm{NT}}+\;e_{\mathrm{NT}}, \end{array}$$

where (for brevity, trait subscripts are omitted): **f** = vector of fixed effects of farm-year with design matrix **F**; **s** = vector of fixed sex effects with design matrix **S**; **v** = vector of fixed effects of the version number of the method used to calculate meat percentage with design matrix **V**; **w**b_M% on w_ denotes the regression of meat percentage on the weights of the animals, **w**; **n** = vector of fixed effects of the parity of the mother with design matrix **N**; **d** = vector of fixed effects of month of birth with design matrix **D**; **l** = vector of random Normal independently distributed litter effects with design matrix **L**; **p** = vector of random NIID distributed pen effects with design matrix **P**; **a** = vector of random normally distributed animal effect with design matrix **Z** and V(**a**) = **G**_x_σ_**a**_^2^, where **G**_x_ is the relationship matrix calculated by method x (see below for the methods used). Variance components of the random effects were previously estimated in a large Norsvin dataset for regular breeding value estimation using pedigree relationships (see Table 1), and were used as known input parameters in the current study. Thus, the variance of the animal effect was assumed constant and did not depend on the relationship matrix used. The analyses were performed by ASREML [10], using the BLUP option.

The alternative methods used for the breeding value estimation are explained below:

**ABLUP:** the numerator relationship matrix **A** (and its inverse) was set up based on pedigree relationships [11].

**G**_**LA**_**BLUP**: following Luan et al. [7], linkage analysis was used to calculate a relationship matrix **G**_**LAj**_ at every marker position j, which were then averaged over all marker positions j to arrive at the final **G**_**LA**_ matrix. The **G**_**LAj**_ matrices were set up using the approach of Fernando and Grossman [12] based on the segregation probabilities, i.e. the probability of inheriting a paternally or maternally derived allele. The latter probabilities were estimated by the LDMIP software [13]. Computationally, **G**_**LA**_ is the most demanding of the **G** matrices. After running LDMIP, it was necessary to set up a gametic relationship matrix at all positions, j, which requires four times as much computer resources per position than setting up **A**. Calculation of the 38 453 **G**_**LAj**_ matrices was parallelised, but computer memory demands increased linearly with the number of **G**_**LAj**_ matrices that were calculated in parallel, which may limit the degree of parallelisation of the computations.

**GBLUP**: The relationship matrix **G** was constructed using the approach of [6]:

$$G^{*}\;=\;\mathrm{XX}’/\left(2\sum p_{j}\left(1-p_{j}\right)\right),$$

where **X** is a matrix of standardised genotypes, with element *X*_*ij*_ = *I*_*ij*_-2*p*_*j*_ and *I*_*ij*_ being the number of “1” alleles that animal *i* carries for SNP *j*. The LDMIP program [13] was used to estimate genotype probabilities for the ungenotyped animals. These genotype probabilities were used to estimate *I*_*ij*_ in the case of missing genotypes.

Because the genotyped animals cannot predict the genotypes of the ungenotyped animals with certainty, a residual relationship matrix, **R**, must be accounted for, i.e. the relationships of the ungenotyped animals given the genotyped animals [14,15]. Following [16], this residual relationship matrix was calculated using **G**_**LA**_ instead of **A**, *i. e*. **R** = **G**_**LA**11_ - **G**_**LA**12_**G**^- 1^_**LA**22_**G**_**LA**21_, where subscript 2 (1) denotes the (un)genotyped block of animals. This R matrix was added to the elements of G^*^ pertaining to the ungenotyped animals to arrive at the final matrix: **G**.

**G**_**H**_**BLUP**: Haplotype alleles were set up following a suggestion by Mike Goddard (personal communication): starting at SNP position j = 0, Step 1: set j = j + 1 and include SNP j into the haplotype (which is relatively easy since the genotypes were phased by Beagle); repeat this step until the number of haplotype alleles exceeds a fixed number (we used 10); Step 2: output the detected haplotype alleles, and go back to Step 1 to set up the haplotypes for the next segment until the entire chromosome is processed. In contrast to the usual methods for setting up haplotypes, in which haplotype boundaries are pre-set, here the boundaries occur at positions where the number of haplotype alleles expands and exceeds the maximum of 10. When extending the size of the haplotype, a large increase in number of haplotype alleles suggests that we are no longer handling a single haplotype but a combination of two adjacent haplotypes, i.e. such positions form a natural place for a haplotype boundary. The total number of haplotypes formed by this method was 54 303.

In order to analyse these haplotypes by the SNP-based methods and software, the haplotypes were translated into SNPs in the following way. If a haplotype at a particular position had four alleles A, B, C and D, this was translated into four ‘artificial’ SNPs where SNP1 has allele ‘1’ when haplotype A occurred and otherwise ‘0’ , SNP2 has allele ‘1’ when haplotype B occurred and otherwise ‘0’ , SNP3 had allele ‘1’ when haplotype C occurred, etc. The recombination rate between these four artificial SNPs was assumed to be very small (10^-5^). In order to obtain predictions of haplotype alleles for ungenotyped animals, the haplotypes were analysed by LDMIP. Next, the artificial SNP genotypes were translated into a relationship matrix, **G**_H_^*^, following the same procedure as used for GBLUP, to which the same **R** matrix as for GBLUP was added for the ungenotyped animals to arrive at the final matrix **G**_**H**_.

### Evaluation of the accuracy of GS

In order to assess the accuracy of GS, the phenotypic records of the youngest animals born after September 2012 were masked in the analyses, i.e. their phenotypes were set to missing. See Table 1 for the numbers of masked records. Next, these records were predicted using the estimates of the effects in the model. The squared correlation, *ρ*^*2*^, between the predicted and real phenotypic records was calculated and interpreted in terms of explained variance of the records, i.e. a fraction *ρ*^*2*^ of the variance of the records could be explained and the unexplained fraction was (1-*ρ*^*2*^). Similar to how SAS (SAS Institute, Cary NC) interprets the (un)explained sum of squares relative to a model that includes only an overall mean, we interpreted the (un)explained variance relative to a model that includes all non-genetic effects, i.e. all effects except the animal effect. If the fraction of the explained variance achieved by a model including only non-genetic effects is denoted by *ρ*_*0*_^*2*^, the extra variance explained by the animal effect in model x, *r*_*x*_^*2*^, was obtained from:

(1) $$\left(1-{\rho_{x}}^{2}\right)\;=\;\left(1-{\rho_{0}}^{2}\right)\;\left(1-{r_{x}}^{2}\right),$$

i.e., the total variance reduction is the product of the variance reduction from the non-genetic model and the variance reduction due to fitting the animal effect.

Since the animal effect cannot explain the environmental variance, the maximum variance reduction is (1 - *ρ*_*max*_^2^) = *σ*_*e*_^*2*^/*V*(*y*), where *σ*_*e*_^*2*^ is from Table 1, and V(*y*) is the variance of the masked records. Using equation (1), this variance reduction was put relative to a model that already contains all non-genetic effects, resulting in *r*_*max*_^*2*^. Note that *r*_*max*_^*2*^ is probably over-estimated because its derivation assumed that not only the animal effects are predicted with an accuracy of 1, but also all other non-genetic effects. Finally, the accuracy of the prediction of the animal effects using model x, i.e. the correlation between predicted and true values, was calculated as *r*_*GSx*_ = *r*_*x*_/*r*_*max*_, which is expected to be underestimated because of the over-estimation of *r*_*max*_.

### Significance testing

The increase in the accuracy of prediction when using a more sophisticated relationship matrix was tested for its statistical significance. A more powerful test can be devised than just considering the standard errors of correlation estimates (which tests whether any two correlation estimates differ), since all correlation estimates were based on the same set of phenotypes. Let $y_{i},\;\hat{y_{1i}}$, and $\hat{y_{2i}}$, denote the recorded phenotype value, its prediction using method 1, and its prediction using method 2 for record *i*, then the correlations between $y_{i},\;\hat{y_{1i}}$, and $\hat{y_{2i}}$, were estimated using the model:

$$\left[\begin{array}{c} y_{i}\\ \hat{y_{1i}}\\ \hat{y_{2i}} \end{array}\right]=\left[\begin{array}{c} \mu_{0}\\ \mu_{1}\\ \mu_{2} \end{array}\right]+\left[\begin{array}{c} e_{0i}\\ e_{1i}\\ e_{2i} \end{array}\right],$$

where [*μ*_0_ *μ*_1_ *μ*_2_] ‘denotes the mean, and [*e*_0*i*_*e*_1*i*_*e*_2*i*_]’ denotes the residuals, which were assumed to have (co)variance matrix:

$$\text{var}\left(\left[\begin{array}{c} e_{0i}\\ e_{1i}\\ e_{2i} \end{array}\right]\right)=\left[\begin{array}{ccc} \sigma_{0}^{2} & r_{01}\sigma_{0}\sigma_{1} & r_{02}\sigma_{0}\sigma_{2}\\ r_{01}\sigma_{0}\sigma_{1} & \sigma_{1}^{2} & r_{12}\sigma_{1}\sigma_{2}\\ r_{02}\sigma_{0}\sigma_{2} & r_{12}\sigma_{1}\sigma_{2} & \sigma_{2}^{2} \end{array}\right]$$

where all variances and correlations were estimated by ASREML [10], together with the log likelood of this alternative-hypothesis model, *LogL*_*1*_. In the null-hypothesis model, the correlation between y_i_ and $\hat{y_{1i}}$ was assumed equal to that between y_i_ and $\hat{y_{2i}}$, i.e. the restriction was *r*_01_ = *r*_02_, which resulted in the likelihood of the null model: *LogL*_*0*_. Under the null-hypothesis, 2(*LogL*_*0*_- *LogL*_*1*_) is approximately chi-squared distributed with one degree of freedom. The resulting P values were halved here because a one-sided test was performed (a priori one of the methods was assumed superior, and if the data did not support this assumption, the test was always considered not-significant). This significance test was applied within one cohort of the youngest animals using another (older) cohort of training animals, and thus does not account for extra variability, e.g. due to different relationships between animals, that occurs if the design would have been replicated.

## Results

Table 2 shows the relationships between the off-diagonal elements of the relationship matrices, since the off-diagonals are most important for the accuracy of breeding value prediction. The variances of the relationships increase as we go from **A** to **G**_**LA**_ to **G**_**H**_ to **G**, where **G** shows 2.3 fold the variance of **A**. This is expected as the relationship matrices make an increasing use of the marker data in this order. The regression coefficient of **G**_**LA**_ on **A** is equal to 1.001, which is expected since both are known to be unbiased estimators of relationships. For the regressions of **G**_**H**_ and **G** on **A**, the regression coefficients are slightly smaller than 1, showing that genetic differences picked up by **A** are also reflected in **G** and **G**_**H**_, with only minor scaling differences (however the regression of **A** on **G** is less than 1 and differences in genomic relationships are not fully reflected in **A**). These minor scaling differences suggest that the use of the same animal variance for all **G** matrices is justified. The regression of **G**_**H**_ and **G** on **G**_**LA**_ are similar to those on **A**. Correlations between the relationships can be as low as 0.6, which shows that pedigree and genomic relationships are quite different; correlations are lower than those found in dairy cattle [7]. The correlations between the elements of **G**_**LA**_ and **A** are high (0.94).

**Table 2:** **Correlations (below the diagonal), variances (on the diagonal), and regression coefficients (B; above the diagonal) of the off-diagonal elements of the different relationship matrices**^**1**^

	**A**	**G**_**LA**_	**G**_**H**_	**G**
A	**0.00129**	1.001	0.925	0.922
G_LA_	0.944	**0.00145**	0.942	0.967
G_H_	0.709	0.765	**0.00219**	1.076
G	0.612	0.680	0.932	**0.00292**

^1^Regression coefficients, B_j on i_, are from the column variable j on the row variable i; the covariance of variables i and j can be calculated as B_j on i_ times the diagonal of i.

Table 3 shows the raw correlations, ρ, between the masked records and their predicted values using the alternative relationship matrices. Generally, there is a substantial improvement in accuracies when moving from the **A** matrix to genomic relationship matrices. Although the improvement due to the implementation of the linkage analysis matrix, **G**_**LA**_, is more moderate, it is statistically significant. Moving from **G**_**LA**_ to **G** yields in most cases a clear and significant improvement. There is a tendency for the **G**_**H**_ matrix to improve the accuracies for two (GR and M%) of the four traits analyzed, but these differences are not significant. It is notable that prediction accuracies are lowest for growth, despite its quite high heritability. This is probably because growth has historically been a major part of the breeding goal, which reduces the between-family genetic variance for this trait, and the between-family component is easier to predict than the within-family (Mendelian sampling) component.

**Table 3:** **Accuracy of prediction of the masked records,***ρ***, for the analysed traits using different relationship matrices**^**1**^

**Trait**	**A**	**G**_**LA**_	**G**	**G**_**H**_
GR	0.136	0.192***	0.294***	0.307^-^
M%	0.265	0.304*	0.468***	0.475^-^
W3W	0.447	0.459**	0.466^-^	0.465^-^
NT	0.284	0.322*	0.420***	0.421^-^

^1^The increase in accuracy when moving from method/column i-1 to i was tested for its statistical significance using a one-sided test where *, **, and *** denote P values < 0.05, < 0.01, and < 0.001, respectively, and – denotes no-significant increase.

Table 4 shows the accuracies of selection, *r*_*GSx*_, for the alternative relationship matrices. Apart from growth (which is commented above), the genomic selection accuracies range from 0.3 to 0.65, which are quite high, especially when considering the fact that candidates have masked phenotypes, the number of genotyped animals is low and the data consist of phenotypic records instead of accurate deregressed proofs. The pattern of the accuracies is similar to that for the correlations in Table 3. For W3W, the accuracy of traditional selection is quite high and is relatively not much improved by genomic selection. Possibly the assumption that W3W is determined only by the genetics of the piglet is not valid, in that the genetics of the mother may also play an important role, but the genotype of the dam was unknown in the current analysis. The **G**_**LA**_ matrix yields substantially less accuracy than the IBS-based relationship matrices.

**Table 4:** **Accuracy of genomic selection, *r***_***GS***_**, for the analysed traits using different relationship matrices**

**Trait**	**A**	**G**_**LA**_	**G**	**G**_**H**_
GR	0.126	0.213	0.353	0.370
M%	0.199	0.299	0.609	0.620
W3W	0.329	0.431	0.487	0.475
NT	0.439	0.499	0.650	0.651

## Discussion

Genomic selection may be seen as a form of traditional BLUP selection where the pedigree relationship matrix, **A**, is substituted by a (more accurate) genomic relationship matrix. Our hypothesis was that genomic relationship matrices based on the IBS of single SNPs may put the base population too far back in time, especially because the SNP panels are often selected for high MAF. Identities at QTL alleles may be due to more recent common ancestors because natural and artificial selection may have eroded ancient genetic differences. The results in Table 2 suggest that we have succeeded in creating relationship matrices that increasingly consider old relationships in the order **A**, **G**_**LA**_, **G**_**H**_ and **G**, since the variance of relationships increases in this order, probably due to considering more old relationships. These more variable relationships are real in the sense that the regression on younger relationships is close to 1 and they result in higher prediction accuracies. Although, there was a tendency for the haplotype-based relationship matrix, **G**_**H**_, to yield higher prediction accuracy than the single-marker based matrix **G** (for two of four traits), these results were not statistically significant. Interestingly, the traits for which **G**_**H**_ tended to yield higher accuracy than **G** (GR and M%) are more heavily selected in pig breeding than the other traits (W3W and NT), suggesting that the use of more recent relationship matrices than **G** is beneficial for more heavily selected traits. However, the **A** and **G**_**LA**_ matrices apparently resulted in relationships that were too young.

The **G**_**H**_ matrix was based on haplotypes with an average size of 8.4 SNPs (result not shown). With a median inter-marker distance of 28 kb [http://res.illumina.com/documents/products/datasheets/datasheet_porcinesnp60.pdf] and assuming a recombination rate of 1 cM per 1000 kb, the recombination rate over a 8.4 SNP region is *θ* ≈ 0.0022, resulting in a base population that occurred approximately 1/(2*θ*) = 223 generations ago [13]. The effective population size of Norwegian Landrace is about *N*_*e*_ = 100 (personal communication Dan Olsen, Norsvin, 2013), which yields an expected heterozygosity of the haplotypes of [17]:

$$\text{Het}=\frac{4N_{e}\theta }{1+4N_{e}\theta }=0.47.$$

Since this expected heterozygosity is close to 50%, the information contained in the haplotypes is close to maximum. The use of larger haplotypes with a higher recombination frequency would not maximise the information contained in the haplotypes but would point to a quite recent common ancestor in the case of haplotype homozygosity, which would be useful when trying to trace young mutations (e.g. disease mutations). Setting the base population to about 200 generations ago agrees also with the history of modern European pig breeds, which originate from a hybridisation with Asian breeds in the 18^th^ or early 19^th^ century [18].

The linkage analysis matrix **G**_**LA**_ yielded poorer prediction accuracies than the **G** matrix (Table 3), which is contrary to the results reported for dairy cattle [7]. The poorer results of linkage analysis in pigs relative to dairy cattle may be because: (1) there was only about one generation of genotyped and phenotyped animals, leaving little opportunity for tracing chromosome segments from one generation to the next by linkage analysis; (2) the information content of phenotypic records is lower than that of deregressed proofs, which combined with the fact that linkage analysis requires re-estimation of chromosomal effects within families, results in a relatively low $r_{GS_{G_{\mathrm{LA}}}}$ for the pig data; (3) the porcine genome sequence map may not be as accurate as its bovine counterpart, which may have hampered the linkage analysis; (4) the *N*_*e*_ of the recent pig population may be larger than that of cattle, which implies that older ancestors and thus relationships are important; (5) the prediction of deregressed proofs in dairy cattle may not require such old relationships compared to prediction of phenotypic records, because deregressed proofs are themselves predicted by a linear model using only recent relationships; (6) the aforementioned hybridisation with Asian breeds [18] will have caused considerable LD, which predates pedigree recording and thus is not captured by **G**_**LA**_. Explanation (5) does, however, not explain the results in Table 2, where the correlations between the relationships in the **G**_**LA**_ and **G** matrices are lower than found by [7] in dairy cattle. In our view, explanations (1) and (2) are the most likely, also if one considers that the youngest animals that were predicted were rarely sibs of the phenotyped animals, due to the high turn-over rate of the elite boars in pig breeding. Moreover, dairy cattle results also indicated that several generations of linkage analysis are needed for high $r_{GS_{G_{\mathrm{LA}}}}$[7].

Several authors have attempted to fit haplotypes for genomic prediction [19-23], mainly based on the argument that haplotypes may show stronger LD with QTL than single SNPs. Here, we used the argument that haplotypes trace younger relationships than the old relationships traced by single SNPs. However, these two arguments are equivalent, which is similar to the equivalence of SNP-BLUP genomic selection with GBLUP [3,24]. Our results also agree with those of [19-23], i.e. the use of haplotypes increases accuracy only sometimes and not by much.

In machine-learning, prediction is viewed as striking a balance between bias and error variance [25], where a model with strong (oversimplifying) assumptions is biased and a more realistic model fits too many effects and thus has large prediction error variances. Haplotype-based models may reflect the LD structure or the relevant age of the relationships better, but they usually fit more effects and, in a situation with limited numbers of training records, may not prove to be more accurate than GBLUP. The SNPs in GBLUP predict relationships over long genetic distances, which reduces the prediction error variance and increases bias but apparently not to the point that the haplotype models are clearly favoured. In the future, the numbers of genotyped animals will increase, which will reduce prediction error variances, and in sequence data, the number of haplotypes is lower than the number of SNPs. All this will favour models that fit haplotype effects.

Deregressed proofs (but the same holds for daughter-yield-deviations or other estimates that more accurately reflect the genetics of an animal than a phenotypic record) are derived from a linear model including the masked and unmasked records. If we write the deregressed proof as DP = TBV + ERR, where TBV is true breeding value, the error term, ERR, is partly due to unmasked records in the training data, and thus can be predicted by the genomic selection model (especially when it is similar to the model that was used to calculate the DP). Hence, when predicting masked DP, predicted accuracies of GS are biased because

$$\begin{array}{ll} \text{Cov}\left(\text{GEBV},\mathrm{DP}\right)\;= & \;\text{Cov}\left(\text{GEBV},\text{TBV}\right)\\ & \;+\;\text{Cov}\left(\text{GEBV},\text{ERR}\right), \end{array}$$

where GEBV denotes genomic breeding value estimates. I.e. the second term inflates the covariance between GEBV and masked DP above that due to prediction of TBV by GEBV (assuming that the errors of the GEBV are positively correlated with the errors from the model that predicts the DP). In this study, we predicted masked phenotypic records. Errors in phenotypic records, i.e., the environmental effects, cannot be predicted by genomic selection, and thus a high accuracy of prediction of records can only be achieved by a high accuracy of prediction of TBV. Thus, when predicting masked phenotypes, the predicted accuracies are not expected to be biased, in contrast to the use of DP or daughter-yield-deviations.

Especially for the analyses of W3W and NT, the data included many phenotypic records on non-genotyped animals (Table 1). Genomic prediction analyses for such data are usually performed by the so-called one-step approach [26,27]. We used an alternative approach here, because of bias problems with the one-step approach [16,28]. For instance, a relationship of 0.55 between full sibs can be explained by linkage analysis, i.e. the sibs happened to inherit more chromosome segments in common from their parents than expected. However the one-step approach explains such increased relationships between family members by adapting the relationships between the founder animals. The latter is because it leaves the regression of ungenotyped onto genotyped animals unaltered (in contrast to linkage analysis). The use of relationship matrix **G**_**LA**_ instead of **A** solves this problem, because in the **G**_**LA**_ matrix these regressions are altered by the marker information [16]. Following [29], we used the estimated genotype probabilities to calculate relationships of ungenotyped animals. In contrast to the one-step approach, this does not require quantification of the difference between a **G** and **A** matrix (also not on the inverse scale), and this avoids scaling problems associated with the one-step approach (e.g. [16]; although the **G**, **G**_**LA**_ and **A** matrices had quite similar scales here; see Table 2). Matrix **R** was added to account for unexplained relationships, but this increased the accuracy of prediction of records only by up to 0.01 (result not shown). The largest increase was for NT, which had most phenotypes recorded on ungenotyped animals. In the future, it is expected that a small minority of phenotypes will come from ungenotyped ancestors, which may make the computationally demanding calculation of the **R** matrix redundant. This will probably require that the diagonal elements of the genomic relationship matrix are calculated by the method of [30] because otherwise they systematically fall below 1 for ungenotyped animals. For ungenotyped descendants of genotyped animals, the one-step method can and should be used, since it is unbiased and optimal for such animals [16].

## Competing interests

The authors declare that they have no competing interests.

## Author’s contributions

THEM performed most of the calculations and wrote the first draft of the manuscript; JO helped developing the methods; IAR collected the phenotypic and pedigree data set and estimated its parameters; EG collected the genomics data and controlled their quality. All authors helped in the writing. All authors read and approved the final manuscript.
